# Association of albumin to globulin ratio with mortality in patients with aneurysmal subarachnoid hemorrhage

**DOI:** 10.1371/journal.pone.0330264

**Published:** 2025-09-22

**Authors:** Yangchun Xiao, Li Li, Yu Zhang, Shiping Chen, Xin Cheng, Wenqing Wang, Yixin Tian, Jialing He, Lu Jia, Chao You, Fang Fang, Dianxiang Lu

**Affiliations:** 1 Department of Neurosurgery, Clinical Medical College and Affiliated Hospital of Chengdu University, Chengdu, Sichuan, China; 2 Department of Neurosurgery, West China Hospital, Sichuan University, Chengdu, Sichuan, China; 3 Department of Neurosurgery, Shanxi Provincial People’s Hospital, Taiyuan, Shanxi, China; Indiana University School of Medicine, UNITED STATES OF AMERICA

## Abstract

**Background:**

The albumin to globulin ratio (AGR) has been associated with poor outcomes in various diseases, but the association with mortality in aneurysmal subarachnoid hemorrhage (aSAH) is still unclear. Our study aimed to explore the relationship between AGR and short-term case fatality in patients with aSAH.

**Methods:**

This retrospective study included 3,421 patients with aSAH at West China Hospital, Sichuan University. All Serum AGRs were obtained within 2 days of admission, and patients were categorized into four groups based on their AGR. Cox regression analyses were conducted to assess the relations between AGR and both 30-day and 90-day mortality.

**Results:**

In 3,421 participants, 339 patients (9.9%) with 30-day mortality, and 407 patients (13.3%) with 90-day mortality. The patients were categorized into four quartiles based on their AGR levels: Q1 (AGR ≤ 1.33), Q2 (AGR: 1.33–1.50), Q3 (AGR: 1.50–1.70), and Q4 (AGR > 1.70). The HR after adjusted for confounders showed significant associations with 30-day mortality, Q2 group (HR 0.72, 95% CI 0.53–0.97), Q3 group (HR 0.72, 95% CI 0.54–0.96), Q4 group (0.53, 95% CI (0.39–0.72)) compared to Q1 group. The association was still significant for 90-day mortality, Q2 group (HR 0.77, 95% CI 0.59–1.01), Q3 group (HR 0.75, 95% CI 0.58–0.98), Q4 group (0.53, 95% CI (0.40–0.71) compared to Q1 group.

**Conclusions:**

A lower admission AGR was significantly associated with an increased risk for short-term case fatality and poor functional outcomes in patients with aSAH. Further prospective research is essential to confirm our findings and explore the mechanisms behind this association.

## Introduction

Aneurysmal subarachnoid hemorrhage (aSAH) is a complex and serious disease with high mortality in China [1] primarily influenced by complications like rebleeding, delayed cerebral ischemia, cerebral vasospasm, and hydrocephalus [2]. Despite the existence of various clinical grading systems such as Hunt and Hess grade [3], World Federation of Neurosurgical Societies (WFNS) [4], the Subarachnoid Hemorrhage International Trialists [5], and Fisher grade [6] aimed at predicting post-aSAH complications and clinical outcomes, their predictive accuracy is limited. Therefore, it is essential to explore additional dependable predictors of aSAH outcomes.

Recent studies have emphasized the significant impact of immunosuppression and inflammation on the progression and results of aSAH [7–11]. In this context, the serum albumin to globulin ratio (AGR) has emerged as a notable measure that reflects both inflammation levels and nutritional health status [12,13]. Serum albumin, as a well-known indicator of nutritional status [13] and inflammation [14–16], played a crucial role in determining patient outcomes [17,18], Conversely, elevated serum globulin levels were associated with increased inflammation severity [19–21]. The integration of serum albumin and globulin levels into the AGR may provide a better predictive value of outcomes in patients with aSAH.

Previous studies have recognized AGR as a valuable predictive biomarker in various diseases like infection, cancer, severe liver disease, and stroke [22–25]. However, its specific relationship with outcomes in aSAH patients remains uncertain. Our study aimed to explore the relationship between AGR and short-term case fatality in patients with aSAH.

## Methods

### Study design

Data from all patients diagnosed with aSAH and admitted to the Departments of Neurosurgery at West China Hospital, Sichuan University, over nine years (between 1 January 2009, and 31 July 2019) were systematically collected. The data collection process was completed in August 2022. Information about patient survival was retrieved from the Household Registration Administration System of the People’s Republic of China. Our study at West China Hospital received approval from the local ethics committee. Informed consent was waived as the study was a clinical audit. We adhered to the STROBE criteria and followed the ethical guidelines of the 1964 Declaration of Helsinki.

### Patient selection

In our study, all participants were confirmed to have aSAH, with the diagnosis of intracranial artery aneurysm and the location of aneurysms established through computed tomography angiography, magnetic resonance angiography, or digital subtraction angiography upon admission and subsequently evaluated by 2 neurologists during hospital stay. The focus of this study was on individuals aged 18 years and older.

The following exclusion criteria were applied: patients with subarachnoid hemorrhage caused by traumatic brain injury, rupture of vascular malformation, or coagulation abnormalities that were not related to primary aSAH [2]; Absence of serum globulin or albumin data within 48 hours of admission [3]; The absence of patient’s information of survival from the Household Registration Administration System due to the unavailability of residents’ identification numbers [4]. Patients with pre-admission infections.

### Clinical characteristics and laboratory data collection

Demographic factors (such as age, sex, smoking, and alcohol), pre-existing medical conditions or diseases (such as hypertension, anticoagulants or antiplatelet drugs usage, diabetes, chronic renal failure, chronic heart disease, chronic liver disease, chronic obstructive pulmonary disease), clinical and radiological characteristics (size and location of the aneurysm), admission status (World Federation of Neurosurgical Societies grade, Fisher grade, Hunt and Hess grade, systolic blood pressure), and laboratory parameters within 24 hours of admission (platelet calculation, activated partial thromboplastin time, glucose level, neutrophil calculation, globulin level, albumin level, AGR) were collected. Aneurysm treatments included clipping, coiling, or no treatment (S1 Definitions for all independent variables).

### Exposure

Within the first 24 hours of admission, we gathered all admission AGR. Subsequently, the patients were classified into four quartiles according to their AGR levels: Q1 (AGR ≤ 1.33), Q2 (AGR: 1.33–1.50), Q3 (AGR: 1.50–1.70), and Q4 (AGR > 1.70).

### Outcomes

The primary endpoint in our study was short-term case fatality, covering both 30-day and 90-day mortality rates. Mortality data were obtained from the Household Registration Administration System of China [26]. Functional status at discharge was evaluated with the modified Rankin Scale (mRS), and poor functional outcomes were defined as an mRS score ranging from 4 to 5.

In-hospital complications were defined as follows: Acute kidney injury diagnosed based on serum creatinine elevation ≥0.3 mg/dL within 48 hours or ≥1.5 times baseline within 7 days, or urine output <0.5 mL/kg/h for 6 hours [27]. Hospital-acquired pneumonia was defined as a new pulmonary infiltrate occurring at least 48 hours after hospital admission [28]. Hospital-acquired infections were identified according to standard clinical and microbiological criteria used in our institution [28]. Delayed cerebral ischemia was diagnosed based on new neurological deficits or radiological evidence of cerebral infarction not attributable to other causes [2,29]. Hydrocephalus is diagnosed by clinical signs and radiographic evidence of ventricular enlargement requiring intervention [2,29]. Rebleeding is defined as a new hemorrhage confirmed by imaging after initial aneurysm rupture, before definitive aneurysm treatment [2,29]. Seizures Clinical seizures were identified based on neurological examination and electroencephalographic confirmation when available, following standard neurological diagnostic criteria [2,29].

### Statistical analysis

The statistical analyses for baseline characteristics and laboratory parameters were performed with R software (version 4.3.2; R Foundation for Statistical Computing). Patients were categorized based on AGR. Two-sided statistical tests were conducted with a significance level set at a P value less than 0.05. Continuous data were assessed through the student’s t-test and Mann-Whitney U-test, while categorical data were evaluated with either the chi-square test or Fisher’s exact test. Missing data were managed using multiple imputation techniques.

In examining independent prognostic variables for short-term case fatality, Cox regression was utilized for both univariate and multivariate analyses. Likewise, Logistic Regression was utilized to explore independent prognostic factors for poor functional outcomes and complications in both univariate and multivariate analyses. Unadjusted and adjusted odds ratios (ORs) or hazard ratios (HRs) along with their corresponding 95% confidence intervals (CIs) were computed. The variables chosen encompassed age, sex, smoking history, alcohol consumption, hypertension, diabetes, chronic obstructive pulmonary disease, chronic renal failure, chronic liver disease, chronic heart disease, aneurysm size, aneurysm location, Fisher grade, aneurysm treatment method, use of External ventricular drain, Hunt and Hess grade, blood glucose levels, and neutrophil count, based on existing research findings and clinical expertise [30].

The study analyzed the HR related to short-term case fatality risk in aSAH patients concerning AGR utilizing restricted cubic spline analysis, with the Q1 group of serum AGR serving as the reference. Collinearity assessment in the multivariate regression analysis was conducted utilizing the variance inflation factor. Survival curves were constructed through the Kaplan-Meier method, and comparisons were made employing the log-rank test.

The diversity of outcomes was assessed through subgroup analyses that considered a range of demographic and clinical factors. Factors under consideration encompassed age categories (over 65 years or not), smoking or not, alcohol consumption, hypertension, diabetes, chronic liver disease, chronic heart disease, chronic obstructive pulmonary disease, systolic blood pressure, aneurysm size, and location, as well as Hunt and Hess, and Fisher grades.

## Results

**Fig 1:**
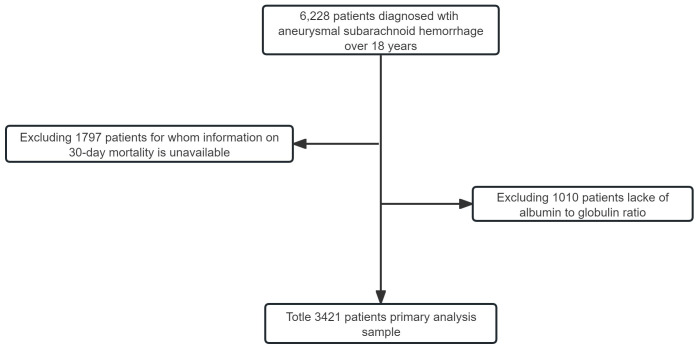
Flow chart of enrollment.

A total of 6,228 patients with aSAH and more than 18 years at West China Hospital were taken in our study (Fig 1). Of these, 1,979 patients were excluded for the loss of survivor information recorded in the Household Registration Administration System, and 1,010 patients were excluded for lacking AGR upon admission. Ultimately, this study involved 3,421 aSAH patients (Supplemental Material 2), out of which there were 339 patients (9.9%) who experienced 30-day case fatality and 407 patients (13.3%) who experienced 90-day case fatality. The baseline characteristics of the groups are detailed in Table 1. Patients with higher AGR levels tended to be younger, more male, more likely to consume alcohol, and less prone to suffer from chronic obstructive pulmonary disease. Moreover, these patients with higher AGR levels seem to have better clinical status on admission and lower Hunt & Hess grades. We have compared the baseline characteristics between excluded and included patients in S1 Table in S1 File. This comparison shows some differences exist, such as Fisher grade, operation, and Hunt and Hess grade.

**Table 1:** Baseline characteristics of the patients.

Characteristics	Albumin to Globulin Ratio					P	Miss value (n, %)
	Overall (n = 3421)	Q1 (≤1.33, n = 890)	Q2 (1.33-1.5, n = 844)	Q3 (1.5-1.7, n = 852)	Q4 (>1.7, n = 835)		
Demographics							
Age, years, mean (SD)	55.13 (12.00)	56.71 (11.95)	55.57 (11.85)	54.61 (11.55)	53.53 (12.43)	<0.001	
Female, n (%)	2220 (64.9)	638 (71.7)	602 (71.3)	542 (63.6)	438 (52.5)	<0.001	
Smoking, n (%)	147 (4.3)	31 (3.5)	33 (3.9)	39 (4.6)	44 (5.3)	0.281	
Alcohol, n (%)	680 (19.9)	147 (16.5)	134 (15.9)	187 (21.9)	212 (25.4)	<0.001	
Medical history, n (%)							
Hypertension	845 (24.7)	232 (26.1)	215 (25.5)	202 (23.7)	196 (23.5)	0.514	
Diabetes	194 (5.7)	59 (6.6)	31 (3.7)	61 (7.2)	43 (5.1)	0.008	
Chronic Obstructive Pulmonary Disease	248 (7.2)	84 (9.4)	52 (6.2)	55 (6.5)	57 (6.8)	0.031	
Chronic renal Failure	21 (0.6)	6 (0.7)	4 (0.5)	7 (0.8)	4 (0.5)	0.759	
Chronic liver disease	300 (8.8)	86 (9.7)	65 (7.7)	81 (9.5)	68 (8.1)	0.38	
Chronic heart disease	85 (2.5)	29 (3.3)	21 (2.5)	21 (2.5)	14 (1.7)	0.217	
SBP, mmHg, mean (SD)	144.4 (25)	145.90 (25)	144.5 (25)	143.4 (25)	143.8 (25)	0.165	25 (0.73)
Aneurysm characteristics							
Size of aneurysm, cm, mean (SD)	0.77 (0.70)	0.76 (0.63)	0.76 (0.66)	0.76 (0.72)	0.79 (0.80)	0.847	772 (22.6)
Anterior circulation aneurysm, n (%)	2791 (81.6)	753 (84.6)	708 (83.9)	684 (80.3)	646 (77.4)	<0.001	
Fisher grade, n (%)						0.025	
I	148 (4.3)	40 (4.5)	48 (5.7)	32 (3.8)	28 (3.4)		
II	522 (15.3)	138 (15.5)	132 (15.6)	149 (17.5)	103 (12.3)		
III	392 (11.5)	90 (10.1)	93 (11.0)	98 (11.5)	111 (13.3)		
IV	1450 (42.4)	382 (42.9)	331 (39.2)	360 (42.3)	377 (45.1)		
Miss	909 (26.6)	240 (27.0)	240 (28.4)	213 (25.0)	216 (25.9)		
Hunt & Hess grade, n (%)						0.575	
I-III	3371 (98.5)	875 (98.3)	829 (98.2)	843 (98.9)	824 (98.7)		
IV-V	50 (1.5)	15 (1.7)	15 (1.8)	9 (1.1)	11 (1.3)		
Treatment of aneurysms, n (%)						0.433	
Clip	2287 (66.9)	583 (65.5)	568 (67.3)	579 (68.0)	557 (66.7)		
Coil	432 (12.6)	108 (12.1)	110 (13.0)	116 (13.6)	98 (11.7)		
No treatment	702 (20.5)	199 (22.4)	166 (19.7)	157 (18.4)	180 (21.6)		
External ventricular drain, n (%)	73 (2.1)	29 (3.3)	7 (0.8)	14 (1.6)	23 (2.8)	0.002	
Laboratory tests, mean (SD)							
Platelet, 10^9^/L	174.4 (70.5)	186.5 (81.8)	178.2 (75.5)	168.0 (62.0)	164.7 (57.8)	<0.001	242 (7.07)
Activated partial thromboplastin time, s	1.03 (0.16)	1.06 (0.17)	1.03 (0.21)	1.02 (0.14)	1.01 (0.11)	<0.001	476 (13.9)
Blood glucose, mmol/L	7.00 (2.44)	7.06 (2.43)	6.84 (2.20)	7.04 (2.57)	7.06 (2.54)	0.198	1 (0.03)
Neutrophil count, 10^9^/L	8.85 (4.47)	8.63 (4.15)	8.65 (4.50)	8.92 (4.51)	9.20 (4.70)	0.036	243 (7.10)
Globulin, g/L	26.85 (4.56)	30.83 (4.81)	27.91 (3.13)	25.73 (2.56)	22.67 (2.70)	<0.001	
Albumin, g/L	39.85 (5.23)	35.95 (5.43)	39.63 (4.36)	41.14 (4.05)	42.93 (4.16)	<0.001	
Albumin to Globulin Ratio, mean (SD)	1.52 (0.29)	1.17 (0.14)	1.42 (0.05)	1.60 (0.06)	1.91 (0.18)	<0.001	

SBP: systolic blood pressure.

AGR: Albumin to Globulin Ratio.

The study’s primary outcomes were analyzed through multivariate Cox regression, as displayed in Table 2. The HR after adjusting for potential confounders, revealed significant associations with 30-day mortality, the Q2 group (HR 0.70, 95% CI 0.52–0.95), Q3 group (HR 0.72, 95% CI 0.54–0.96) Q4 group (0.54, 95% CI (0.39−0.73)) compared to Q1 group. The association was still significant for 90-day mortality, Q2 group (HR 0.72, 95% CI 0.59–0.89), Q3 group (HR 0.70, 95% CI 0.54–0.92), Q4 group (0.54, 95% CI (0.41–0.72) compared to Q1 group. The confounders taken in the multivariable Cox regression analysis were shown in S2 Table in S1 File. The adjusted HR for 90-day mortality was also significant (S3 Table in S1 File).

**Table 2:** The association between albumin to globulin ratio and mortality using multivariate Cox regression.

Outcomes		Events/Total, n (%)	Cox regression Unadjusted HR	P	Cox regression Adjusted HR*	P
30-day mortality						
Continues		339/3421 (9.9%)	0.47 (0.32-0.68)	<0.001	0.51 (0.36-0.72)	<0.001
Dichotomy	Low AGR (≤1.50)	190/1734 (11%)	1 [Reference]		1 [Reference]	
	High AGR (>1.50)	149/1687 (8.8%)	0.79 (0.64- 0.98)	0.035	0.72 (0.57-0.90)	0.004
Quartiles	Q1 (AGR ≤ 1.33)	118/890 (13.3%)	1 [Reference]		1 [Reference]	
	Q2 (AGR:1.33–1.50)	72/844 (8.5%)	0.63 (0.47- 0.84)	0.002	0.70 (0.52-0.95)	0.021
	Q3 (AGR:1.50–1.70)	82/852 (9.6%)	0.71 (0.53- 0.94)	0.016	0.72 (0.54-0.96)	0.025
	Q4 (AGR > 1.70)	67/835 (8%)	0.59 (0.44- 0.79)	0.001	0.54 (0.39-0.73)	<0.001
90-day mortality						
Continues		407/3421 (11.9%)	0.48 (0.34-0.67)	<0.001	0.52 (0.38-0.72)	<0.001
Dichotomy	Low AGR (≤1.50)	230/1734 (13.3%)	1 [Reference]		1 [Reference]	
	High AGR (>1.50)	177/1687 (10.5%)	0.78 (0.64- 0.95)	0.012	0.72 (0.59-0.89)	0.002
Quartiles	Q1 (AGR ≤ 1.33)	140/890 (15.7%)	1 [Reference]		1 [Reference]	
	Q2 (AGR:1.33–1.50)	90/844 (10.7%)	0.66 (0.50- 0.86)	0.002	0.72 (0.55-0.95)	0.02
	Q3 (AGR:1.50–1.70)	97/852 (11.4%)	0.70 (0.54- 0.91)	0.008	0.70 (0.54-0.92)	0.01
	Q4 (AGR > 1.70)	80/835 (9.6%)	0.59 (0.45- 0.77)	<0.001	0.54 (0.41-0.72)	<0.001

*The model was adjusted for age, sex, smoking, hypertension, chronic renal failure, chronic liver disease, size of aneurysm, anterior circulation aneurysm, Fisher grade, treatment of aneurysms, Hunt & Hess grade, neutrophil count, and blood glucose.

AGR: Albumin to Globulin Ratio.

In the study, multivariate logistic regression analyses were conducted to examine the association between AGR levels and poor functional outcomes as indicated by mRS scores of 4–5, detailed in Table 3. The ORs remained statistically significant for different AGR quartile groups: Q2 (OR 0.75, 95% CI: 0.56–1.01), Q3 (OR 0.68, 95% CI: 0.51–0.92), and Q4 (HR 0.69, 95% CI: 0.51–0.93), in comparison to the reference Q1 group. Moreover, Table 4 displays the associations between AGR levels and postoperative complications. Elevated AGR levels were found to be associated with the decreasing occurrence of Acute kidney injury, delayed cerebral ischemia, and hydrocephalus in patients with aSAH, with OR of 0.68 (95% CI: 0.48–0.95),0.76 (95% CI: 0.64–0.91) and 0.74 (95% CI: 0.59–0.91) respectively.

**Table 3:** The association between albumin to globulin ratio and poor functional outcomes using multivariate logistic regression.

Characters		Events/Total, n (%)	Logistic Regression Unadjusted OR	P	Logistic Regression Adjusted OR*	P
Poor functional outcomes						
Continues		900/3421 (26.3%)	0.51 (0.38-0.68)	<0.001	0.54 (0.37-0.78)	0.001
Dichotomy	Low AGR (≤1.50)	392/1630 (24%)	1 [Reference]		1 [Reference]	
	High AGR (>1.50)	314/1587 (19.8%)	0.78 (0.66-0.92)	0.004	0.78 (0.63-0.97)	0.027
Quartiles	Q1 (AGR ≤ 1.33)	228/826 (27.6%)	1 [Reference]		1 [Reference]	
	Q2 (AGR:1.33–1.50)	164/804 (20.4%)	0.67 (0.53-0.85)	0.001	0.75 (0.56-1.01)	0.056
	Q3 (AGR:1.50–1.70)	151/802 (18.8%)	0.61 (0.48-0.77)	<0.001	0.68 (0.51-0.92)	0.012
	Q4 (AGR > 1.70)	163/785 (20.8%)	0.69 (0.55-0.87)	0.001	0.69 (0.51-0.93)	0.014

*The model was adjusted for age, hypertension, diabetes, chronic obstructive pulmonary disease, chronic liver disease, size of aneurysm, anterior circulation aneurysm, Hunt and Hess grade, treatment of aneurysms, Hunt & Hess grade, neutrophil count, and blood glucose.

AGR: Albumin to Globulin Ratio.

Poor functional outcomes are defined as a modified Rankin Scale score of 4–5.

**Table 4:** The association between albumin to globulin ratio and complications using multivariate logistic regression.

Characters	Events/Total, n (%)	Logistic Regression unadjusted OR	P	Logistic Regression Adjusted OR*	P
Acute kidney injury	78/1687 (4.6%)	0.85 (0.62-1.15)	0.29	0.81 (0.58-1.12)	0.03
Hospital infections	610/1687 (36.2%)	0.93 (0.81-1.06)	0.28	0.90 (0.77-1.05)	0.19
Pneumonia	428/1687 (25.4%)	0.95 (0.81-1.10)	0.49	0.92 (0.78-1.09)	0.34
Delayed Cerebral Ischemia	277/1687 (16.4%)	0.74 (0.63-0.89)	0.001	0.76 (0.64-0.91)	0.002
Hydrocephalus	156/1687 (9.2%)	0.76 (0.61-0.94)	0.013	0.74 (0.59-0.94)	0.014
Rebleeding	91/1687 (5.4%)	1.34 (0.97-1.84)	0.07	1.22 (0.88-1.69)	0.23
Seizure	53/1687 (3.1%)	0.77 (0.54-1.11)	0.16	0.75 (0.51-1.09)	0.13

The data was dichotomy into the Low AGR group (≤1.50) and High AGR group (>1.50).

*The model was adjusted for age, sex, alcohol, smoking, hypertension, diabetes, systolic blood pressure, size of aneurysm, anterior circulation aneurysm, Fisher grade, treatment of aneurysms, external ventricular drain, Hunt and Hess grade, neutrophil count, and blood glucose.

**Fig 2:**
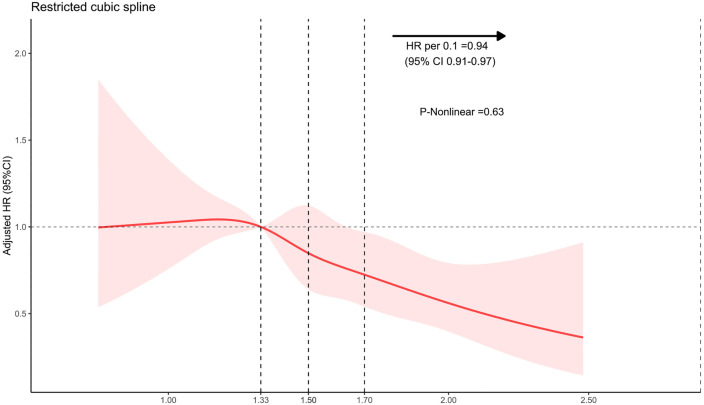
The restricted cubic spline depicting the hazard ratio of albumin to globulin ratio associated with 30-day mortality among patients with aneurysmal subarachnoid hemorrhage. The x-axis represents the Albumin-to-globulin ratio, while the y-axis depicts the hazard ratio of 30-day mortality. The model was adjusted for age, sex, smoking, hypertension, chronic renal failure, chronic liver disease, size of aneurysm, anterior circulation aneurysm, Fisher grade, Hunt & Hess grade, treatment of aneurysms, WFNS grade, neutrophil count, and blood glucose. Red indicates 95% CIs; HR: hazard ratio.

**Fig 3:**
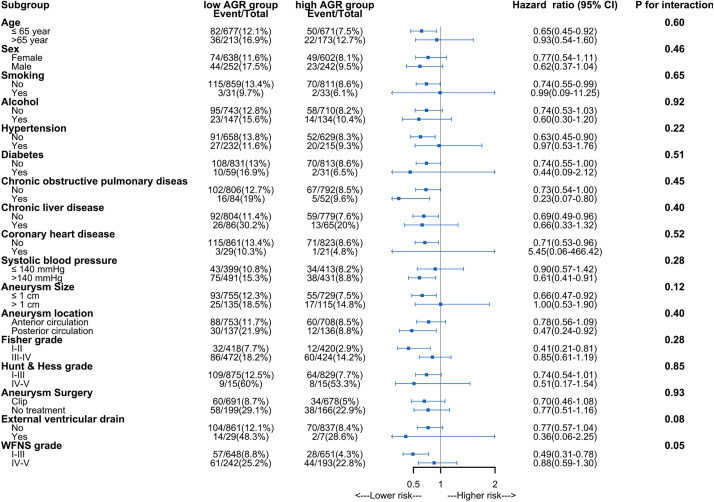
Subgroup analysis of association between albumin to globulin ratio and 30-day mortality. WFNS: World Federation of Neurosurgical Societies.

The result of the Kaplan-Meier survival curves in S1 Fig in S1 File showed that patients with higher AGR levels exhibited increased survival rates compared to patients with lower AGR levels. The application of Restricted Cubic Splines in the analysis revealed a continuous relationship between admission AGR and 30-day mortality, as shown in Fig 2. The results suggest a consistent association between higher AGR values and reduced 30-day mortality. Subgroup analysis results are presented in Fig 3, showing no significant interactions observed among all patients.

## Discussion

In this retrospective cohort study, we observed a significant association between admission AGR levels and adverse outcomes in patients diagnosed with aSAH. Elevated AGR levels upon admission were associated with reduced 30-day and 90-day mortality rates. Moreover, our results suggested a possible association between higher admission AGR levels and a lower incidence of delayed cerebral ischemia and hydrocephalus in aSAH patients.

Wang et al. [22] demonstrated that elevated AGR predicts improved survival and functional outcomes in aSAH patients, aligning with our findings. Similarly, Guo et al. [31] identified systemic inflammatory markers as predictors of post-aSAH complications like delayed cerebral ischemia, consistent with our observed AGR-inverse correlation with such complications. Zhang et al. [32,33] further confirmed that reduced AGR indicates inflammatory/malnutrition states linked to poorer prognosis, reinforcing our conclusions about AGR’s dual role as both nutritional and inflammatory prognostic indicator in aSAH. Consistent with prior studies, our research also illustrates that higher AGR levels are connected to reduced short-term case fatality and improved functional status in patients with aSAH. However, there exist some differences from previous research in various aspects. Our investigation not only scrutinized the relationship between AGR levels and short-term case fatality rates and functional outcomes but also examined the reduced occurrence of complications such as delayed cerebral ischemia and hydrocephalus in aSAH patients.

Albumin has demonstrated antioxidant and anti-inflammatory properties that reduce oxidative stress and neuronal death to protect against secondary brain injuries [34,35]. Another study conducted by Di Napoli et al. demonstrated that hypoalbuminemia is strongly associated with SIRS, a systemic inflammatory state that independently predicts poor functional outcomes and in-hospital mortality in ICH patients [36]. Low albumin levels are associated with complications which may increase mortality in aSAH patients [37–39]. Conversely, serum globulin is commonly recognized as an acute-phase protein, with elevated levels independently associated with higher in-hospital mortality and an increased incidence of in-hospital pneumonia [40]. Hence, in this context, the AGR may perform as a more established biomarker of mortality. Lower AGR was also associated with higher incidences of complications such as pneumonia [41] and delayed cerebral ischemia [31], which contributed to mortality in aSAH patients. Moreover, our analysis also showed that lower AGR was associated with higher incidences of complications such as Acute kidney injury, delayed cerebral ischemia, and hydrocephalus, and increased risks of mortality in aSAH patients.

While our study could not analyze cause-specific mortality, the complications associated with low AGR, such as delayed cerebral ischemia, hydrocephalus, and hospital-acquired infections, are well-documented drivers of mortality in aSAH. For instance, delayed cerebral ischemia predisposes patients to cerebral infarction, a leading cause of death, while hospital-acquired infections increase sepsis risk. Similarly, hydrocephalus contributes to fatal intracranial pressure elevations. The AGR association with these complications suggests it indirectly reflects susceptibility to mortality subtypes via these pathways.

The AGR has been indicated as a valuable biomarker in patients with aSAH by our findings. Clinicians may use the AGR as a prognostic indicator for patients with aSAH, with higher AGR at admission associated with reduced mortality rates. A lower AGR ratio may be linked to an increased occurrence of delayed cerebral ischemia and hydrocephalus, which are significant complications in patients with aSAH. Clinicians can utilize the AGR to risk-stratify patients, tailor treatment strategies, and predict clinical outcomes in patients with aSAH.

Our study has several limitations. First, the overall demographic and clinical profiles are largely comparable in S1 Table in S1 File, suggesting that the excluded patients do not differ markedly from those included in key baseline variables. This reduces but does not eliminate concerns about selection bias (S3 Table in S1 File). Second, the missing data may result from logistical and clinical factors such as incomplete laboratory testing, rather than systematic exclusion based on patient characteristics or prognosis, which could introduce bias. Third, While our current dataset encompasses a large sample size over 10 years, we acknowledge that it lacks supportive care, neurocritical monitoring, detailed temporal stratification, and granular data on intervention timing and level of care changes, limiting our ability to perform such focused subgroup analyses. Fourth, our data lacked information on specific mortality subtypes, and the treatments for complications due to data constraints. While previous studies have noted associations between AGR and outcomes in aSAH patients, the mechanism of this relationship remains inadequately understood. Last, our findings are specific to aneurysmal SAH and may not generalize traumatic or non-aneurysmal SAH subtypes. Hence, it is imperative to conduct further prospective studies to validate our findings and improve our understanding of the correlation between AGR and mortality in this specific patient population.

## Conclusion

A lower admission AGR was significantly associated with an increased risk for short-term case fatality and poor functional outcomes in patients with aSAH. Further prospective research is essential to confirm our findings and explore the mechanisms behind this association.

## Supporting information

S1 File Supplemental Material. (DOCX)

S2 File Renamed_18661. (CSV)

## Abbreviations

AGR: albumin to globulin ratio
aSAH: aneurysmal subarachnoid hemorrhage
WFNS: World Federation of Neurosurgical Societies
mRS: modified Rankin Scale
OR: odds ratio
HR: hazard ratios
CI: confidence interval
