# Supplementary material for: Association of albumin to globulin ratio with mortality in patients with aneurysmal subarachnoid hemorrhage
Source: PLoS One. 2025 Sep 22;20(9):e0330264. doi: 10.1371/journal.pone.0330264 (PMC12453187; doi:10.1371/journal.pone.0330264)
Supplement: S1 File — (DOCX) [file pone.0330264.s001.docx]

**Supporting information 1**

**S1 Definitions for all independent variable**

**S1 Table Baseline Characteristics of the included and excluded Patients.**

**S2 Table Factors Associated with 30-day Mortality for All Patients.**

SBP: systolic blood pressure; WFNS: World Federation of Neurosurgical Societies; NA: not available

**S3 Table Factors Associated with 90-day Mortality for All Patients.**

SBP: systolic blood pressure; WFNS: World Federation of Neurosurgical Societies; NA: not available

**S1 Fig Kaplan-Meier Estimates for 1-year Survival for Patients with Aneurysmal Subarachnoid Hemorrhage.**

Patients were categorized into the four predefined AGR quartiles (Q1–Q4): Q1: AGR ≤ 1.33 (n=890), Q2: AGR 1.33–1.50 (n=844), Q3: AGR 1.50–1.70 (n=852), Q4: AGR >1.70 (n=835)

**S1 Definitions for all independent variables**

Demographic and Clinical Variables:

Age: Patient age at admission in years (continuous variable).

Sex: Male or female as recorded in medical records.

Smoking history: Defined as current or former smoking status reported by the patient or documented in medical history.

Alcohol consumption: Defined as self-reported regular alcohol use prior to admission.

Hypertension: History of hypertension diagnosed by a physician or use of antihypertensive medications before admission.

Diabetes mellitus: Physician-diagnosed diabetes or use of antidiabetic medications prior to admission.

Chronic obstructive pulmonary disease (COPD): Documented diagnosis of COPD in medical history.

Chronic renal failure: History of chronic kidney disease or renal failure documented before admission.

Chronic liver disease: Physician-diagnosed chronic liver disease such as cirrhosis or chronic hepatitis.

Chronic heart disease: History of heart failure, ischemic heart disease, or other chronic cardiac conditions.

Aneurysm Characteristics:

Size of aneurysm: Maximum diameter of the ruptured aneurysm measured in centimeters by computed tomography angiography (CTA), magnetic resonance angiography (MRA), or digital subtraction angiography (DSA) on admission.

Aneurysm location: Categorized as anterior circulation aneurysm (including internal carotid artery, anterior cerebral artery, middle cerebral artery) or posterior circulation aneurysm (basilar artery, vertebral artery, posterior cerebral artery).

Clinical Severity and Grading Scales:

Hunt and Hess grade: Clinical grading scale for aSAH severity ranging from I (asymptomatic or mild headache) to V (deep coma).

Fisher grade: Radiological grading scale based on the amount and distribution of subarachnoid blood on CT scan; grades I to IV.

Systolic blood pressure (SBP): Measured in mmHg on admission.

**S1 Table Baseline Characteristics of the included and excluded Patients.**

| Characteristics | Albumin to Globulin Ratio | | P | SMD |
| --- | --- | --- | --- | --- |
|  | Excluded (n=2807) | Included  (n=2807) |  |  |
| Demographics |  |  |  |  |
| Age, years, mean (SD) | 55.15 (12.5) | 55.13 (12) | 0.95 | 0.002 |
| Female, n (%) | 1812 (64.6) | 2220 (64.9) | 0.8 | 0.007 |
| Smoking, n (%) | 110 (3.9) | 147 (4.3) | 0.5 | 0.019 |
| Alcohol, n (%) | 535 (19.1) | 680 (19.9) | 0.44 | 0.021 |
| Medical history, n (%) |  |  |  |  |
| Hypertension | 722 (25.7) | 845 (24.7) | 0.37 | 0.024 |
| Diabetes | 139 (5.0) | 194 (5.7) | 0.23 | 0.032 |
| Chronic Obstructive Pulmonary Disease | 170 (6.1) | 248 (7.2) | 0.07 | 0.048 |
| Chronic renal Failure | 5 (0.2) | 21 (0.6) | 0.014 | 0.069 |
| Chronic liver disease | 189 (6.7) | 300 (8.8) | 0.003 | 0.076 |
| Chronic heart disease | 99 (3.5) | 85 (2.5) | 0.019 | 0.061 |
| SBP, mmHg, mean (SD) | 146 (26) | 144 (25.) | 0.001 | 0.085 |
| Aneurysm characteristics |  |  |  |  |
| Size of aneurysm, cm, mean (SD) | 0.74 (0.63) | 0.77 (0.70) | 0.17 | 0.04 |
| Anterior circulation aneurysm, n (%) | 473 (16.9) | 630 (18.4) | 0.115 | 0.041 |
| Hunt & Hess grade IV-V, n (%) | 87 (3.1) | 50 (1.5) | <0.001 | 0.11 |
| Fisher grade |  |  | <0.001 | 0.197 |
| I | 84 (3.0) | 148 (4.3) |  |  |
| II | 412 (14.7) | 522 (15.3) |  |  |
| III | 343 (12.2) | 392 (11.5) |  |  |
| IV | 1411 (50.3) | 1450 (42.4) |  |  |
| Miss | 557 (19.8) | 909 (26.6) |  |  |
| Treatment of aneurysms, n (%) |  |  | <0.001 | 0.118 |
| Clip | 1767 (62.9) | 2287 (66.9) |  |  |
| Coil | 325 (11.6) | 432 (12.6) |  |  |
| No treatment | 715 (25.5) | 702 (20.5) |  |  |
| External ventricular drain, n (%) | 69 (2.5) | 73 (2.1) | 0.377 | 0.034 |

**S2 Table Factors Associated with 30-day Mortality for All Patients**

| Characteristics | Univariable | | Multivariable | |
| --- | --- | --- | --- | --- |
|  | HR (95% CI) | P | HR (95% CI) | P |
| Demographics |  |  |  |  |
| Age | 1.02(1.01- 1.03) | <0.001 | NA | NA |
| Female | 0.77(0.62- 0.95) | 0.016 | 0.76(0.60-0.97) | 0.026 |
| Smoking | 0.88(0.50- 1.53) | 0.643 | 0.62(0.35-1.09) | 0.098 |
| Alcohol | 1.12(0.86- 1.45) | 0.408 | NA | NA |
| Medical history |  |  |  |  |
| Hypertension | 1.16(0.91- 1.47) | 0.232 | 0.81(0.63-1.05) | 0.107 |
| Diabetes | 1.46(0.99- 2.17) | 0.057 | NA | NA |
| Chronic Obstructive Pulmonary Disease | 1.21(0.83- 1.77) | 0.33 | NA | NA |
| Chronic renal Failure | 4.47(2.22- 9.02) | <0.001 | 1.81(0.85-3.87) | 0.124 |
| Chronic liver disease | 2.92(2.25- 3.80) | <0.001 | 1.66(1.26-2.19) | 0 |
| Chronic heart disease | 1.08(0.56- 2.10) | 0.818 | NA | NA |
| SBP | 1.01(1.00- 1.01) | <0.001 | 1.00(1.00-1.01) | 0.104 |
| Aneurysm characteristics |  |  |  |  |
| Size of aneurysm | 1.22(1.09- 1.35) | <0.001 | 1.22(1.10-1.37) | 0 |
| Anterior circulation aneurysm | 1.40(1.09- 1.80) | 0.009 | NA | NA |
| Fisher grade |  |  |  |  |
| I | 1 [Reference] |  | 1 [Reference] |  |
| II | 1.43(0.55- 3.73) | 0.469 | 1.07(0.41-2.80) | 0.894 |
| III | 1.51(0.57- 4.03) | 0.407 | 1.17(0.44-3.13) | 0.755 |
| IV | 5.29(2.18-12.82) | <0.001 | 2.39(0.98-5.87) | 0.057 |
| Miss | 1.53(0.61- 3.86) | 0.363 | 1.09(0.43-2.75) | 0.855 |
| Hunt & Hess grade |  |  |  |  |
| I-III | 1 [Reference] |  | 1 [Reference] |  |
| IV-V | 12.58(8.77-18.05) | <0.001 | 3.28(2.22-4.86) | 0 |
| Treatment aneurysms |  |  |  |  |
| No treatment | 1 [Reference] |  | 1 [Reference] |  |
| Clip | 0.26(0.18- 0.38) | <0.001 | 0.35(0.24-0.51) | <0.001 |
| Coil | 0.20(0.16- 0.25) | <0.001 | 0.25(0.20-0.31) | <0.001 |
| External ventricular drain | 3.22(2.07- 5.01) | <0.001 | 1.57(1.00-2.49) | 0.052 |
| Laboratory tests |  |  |  |  |
| Platelet | 1.00(1.00- 1.00) | 0.714 | NA | NA |
| Activated partial thromboplastin time | 1.01(1.00- 1.03) | 0.164 | NA | NA |
| Blood glucose | 1.18(1.15- 1.21) | <0.001 | 1.09(1.05-1.12) | <0.001 |
| Neutrophil count | 1.16(1.14- 1.18) | <0.001 | 1.09(1.07-1.11) | <0.001 |
| Admission Albumin to Globulin Ratio |  |  |  |  |
| Q1(≤1.33) | 1 [Reference] |  | 1 [Reference] |  |
| Q2(1.33-1.50) | 0.63(0.47- 0.84) | 0.002 | 0.70(0.52-0.95) | 0.021 |
| Q3(1.50-1.70) | 0.71(0.53- 0.94) | 0.016 | 0.72(0.54-0.96) | 0.025 |
| Q4(>1.70) | 0.59(0.44- 0.79) | 0.001 | 0.54(0.39-0.73) | <0.001 |

SBP: systolic blood pressure; NA: not available

**S3 Table Factors Associated with 90-day Mortality for All Patients**

| Characteristics | Univariable | | Multivariable | |
| --- | --- | --- | --- | --- |
|  | HR (95% CI) | P | HR (95% CI) | P |
| Demographics |  |  |  |  |
| Age | 1.02(1.01- 1.03) | <0.001 | 1.01(1.00-1.02) | 0.042 |
| Female | 0.81(0.67- 0.99) | 0.04 | NA | NA |
| Smoking | 0.90(0.55- 1.48) | 0.681 | NA | NA |
| Alcohol | 1.12(0.89- 1.42) | 0.336 | 1.35(1.06-1.73) | 0.017 |
| Medical history |  |  |  |  |
| Hypertension | 1.19(0.96- 1.48) | 0.11 | 0.84(0.67-1.05) | 0.127 |
| Diabetes | 1.56(1.10- 2.21) | 0.014 | NA | NA |
| Chronic Obstructive Pulmonary Disease | 1.41(1.02- 1.96) | 0.038 | NA | NA |
| Chronic renal Failure | 5.43(2.98- 9.89) | <0.001 | 2.82(1.49-5.33) | 0.001 |
| Chronic liver disease | 2.95(2.32- 3.75) | <0.001 | 1.82(1.41-2.33) | 0 |
| Chronic heart disease | 1.10(0.60- 2.00) | 0.756 | NA | NA |
| SBP | 1.01(1.01- 1.01) | <0.001 | NA | NA |
| Aneurysm characteristics |  |  |  |  |
| Size of aneurysm | 1.26(1.15- 1.38) | <0.001 | 1.19(1.09-1.30) | <0.001 |
| Anterior circulation aneurysm | 1.33(1.05- 1.67) | 0.017 | NA | NA |
| Fisher grade |  |  |  |  |
| I | 1 [Reference] |  | 1 [Reference] |  |
| II | 1.27(0.56- 2.87) | 0.574 | 0.95(0.42-2.17) | 0.905 |
| III | 1.46(0.64- 3.36) | 0.369 | 1.13(0.49-2.61) | 0.769 |
| IV | 4.54(2.14- 9.60) | <0.001 | 2.07(0.97-4.42) | 0.061 |
| Miss | 1.31(0.60- 2.87) | 0.501 | 0.96(0.44-2.12) | 0.923 |
| Treatment aneurysms |  |  |  |  |
| No treatment | 1 [Reference] |  | 1 [Reference] |  |
| Clip | 0.24(0.17- 0.34) | <0.001 | 0.35(0.24-0.50) | <0.001 |
| Coil | 0.23(0.19- 0.28) | <0.001 | 0.30(0.24-0.37) | <0.001 |
| External ventricular drain | 4.02(2.77- 5.84) | <0.001 | 2.05(1.39-3.02) | <0.001 |
| Laboratory tests |  |  |  |  |
| Platelet | 1.00(1.00- 1.00) | 0.698 | NA | NA |
| Activated partial thromboplastin time | 1.00(1.00- 1.01) | 0.385 | NA | NA |
| Blood glucose | 1.18(1.15- 1.20) | <0.001 | 1.08(1.05-1.12) | 0.001 |
| Neutrophil count | 1.15(1.13- 1.17) | <0.001 | 1.09(1.07-1.11) | <0.001 |
| Admission Albumin to Globulin Ratio |  |  |  |  |
| Q1(≤1.33) | 1 [Reference] |  | 1 [Reference] |  |
| Q2(1.33-1.50) | 0.66(0.50- 0.86) | 0.002 | 0.72(0.55-0.95) | 0.02 |
| Q3(1.50-1.70) | 0.70(0.54- 0.91) | 0.008 | 0.70(0.54-0.92) | 0.01 |
| Q4(>1.70) | 0.59(0.45- 0.77) | <0.001 | 0.54(0.41-0.72) | <0.001 |

SBP: systolic blood pressure; NA: not available


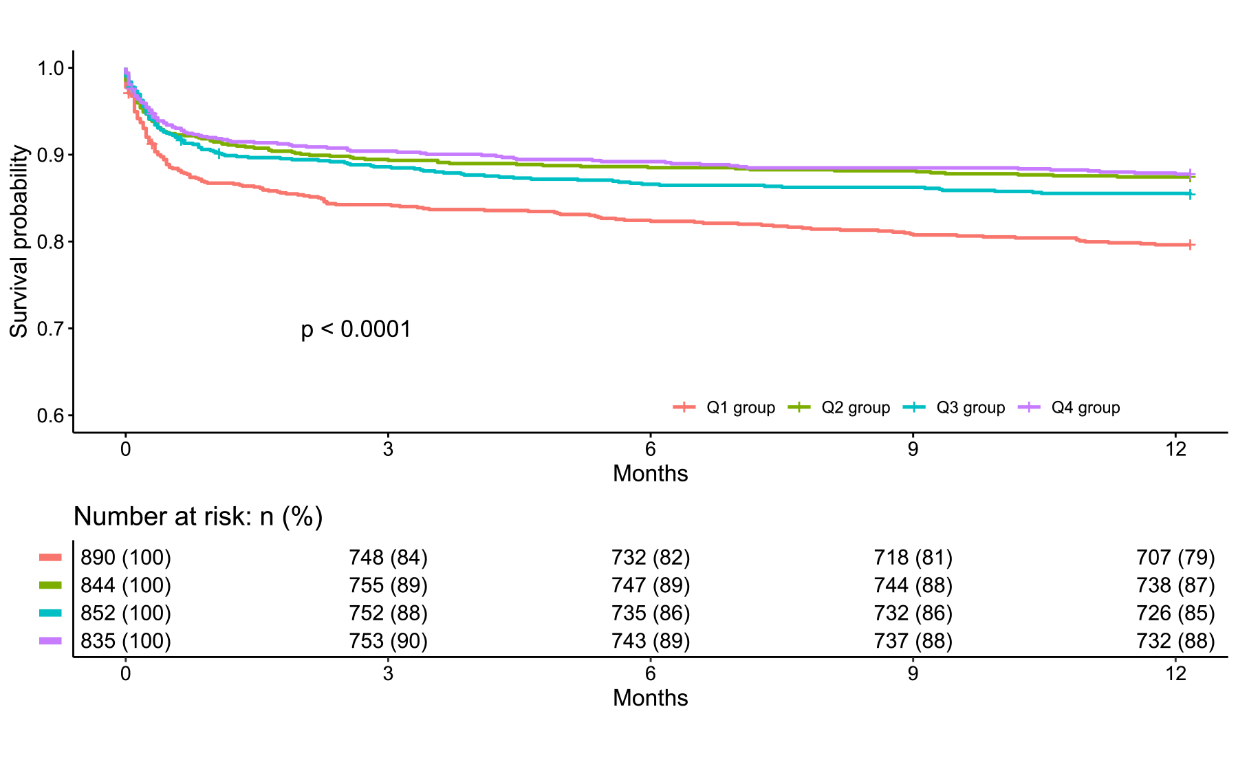


**S1 Fig Kaplan-Meier Estimates for 1-year Survival for Patients with Aneurysmal Subarachnoid Hemorrhage.**

Patients were categorized into the four predefined AGR quartiles (Q1–Q4): Q1: AGR ≤ 1.33 (n=890), Q2: AGR 1.33–1.50 (n=844), Q3: AGR 1.50–1.70 (n=852), Q4: AGR >1.70 (n=835)
